# Fish focus primarily on the faces of other fish

**DOI:** 10.1038/s41598-019-44715-0

**Published:** 2019-06-10

**Authors:** Takashi Hotta, Kento Kawasaka, Shun Satoh, Masanori Kohda

**Affiliations:** 1 Department of Biology and Geosciences, Graduate School of Sciences, Osaka City University, Sumiyoshi, Osaka 558-8585 Japan; 2 Department of Psychology, Graduate School of Letters, Kyoto University, Sakyo, Kyoto 606-8501 Japan

**Keywords:** Animal behaviour, Animal physiology

## Abstract

“Face” is a special stimulus in humans and, nonhuman primates, and some other social mammals; that is, they perceive the face differently from the other body parts and other stimuli. In these species, the face conveys much information, so individuals examine the face at first sight rather than other body parts. Similar to mammals, the faces of fish also convey much information, but little is known about whether fish pay attention to the face or face-viewing patterns. Here we document the face-viewing patterns of the cichlid fish *Neolamprologus brichardi*, which can distinguish between conspecifics based on facial colouration. First, we established a method to identify the point at which subject fish inspected. Fish often fixated in direction to their heads toward the object of attention, suggesting that the extended body axis indicated the attention point. Using this attribute, we examined the point of attention of subject fish presented with photographs of conspecifics and heterospecifics. The results revealed that the fish inspected initially and repeatedly at the face and the duration was longer for the face than other body parts.

## Introduction

How animals view other individuals has received much attention, especially from primatologists^1–5^. Some researchers have examined this issue using ‘eye-tracking’ methods, which are a way to assess gazing behaviours directly^1,5,6^. When humans, apes, and monkeys look at conspecifics, they initially gaze at the face and respond both adequately and immediately after obtaining information on individual identity, motivation, and the focus of attention^1–5^. This indicates that patterns of viewing conspecifics are associated with attention, and the visual cognitive systems direct the gaze toward the important and informative body part^6^.

To our knowledge, little is known on the face-viewing patterns in non-primate animals. However, the faces of animals other than primates also convey much information. For example, mammals generally display a wide range of facial behaviours^7^. Communicative gestures of the head, mouth, and throat have been also reported in some birds^8^, lizards^9^, frogs^10^, and fish^11^. Additionally, the face includes information about individual identity in primates^12^, non-primate mammals^13^, birds^14,15^, fish^16–19^ and paper wasps^20^. Other studies also showed that chicks pecked at head more than at body and feet^21^, and the head and neck region were particularly important for filial preference of neonate chicks^22^. These suggest that face is a special visual stimulus and face-viewing should be common throughout animal kingdom, but there is no evidence of the face-viewing patterns in animals other than primates. Therefore, it is interesting to examine whether non-mammalian vertebrates, such as fish and birds, engage in face-viewing initially similar to primates; if fish do engage in face-viewing, this would be important in terms of understanding the evolution of visual cognitive system and face perception in vertebrates^6^.

We examined how an African cichlid fish *Neolamprologus brichardi* (a sub-species or sister species of *N. pulcher*^23^) views other fish. *N*. *brichardi* is endemic to Lake Tanganyika and is a cooperatively breeding fish that lives in stable social groups that comprise one dominant pair and up to 25 related and unrelated helpers of both sexes^24^. This species can discriminate familiar conspecifics from unfamiliar ones based on facial colouration at the first sight within 0.5 s^16^, and has a black horizontal stripe behind the eye that signals dominance and fighting ability^24–26^, suggesting that the face of *N*. *brichardi* contains important social signals. We hypothesised that these fish have face-viewing patterns similar to those observed in mammals^1–5^. Eye-tracking methods are used when studying face-viewing patterns in humans and primates, but this method is difficult to apply to non-primate vertebrates, especially fish^2,3^. Therefore, we initially explored a method for attention tracking in fish, similar to eye-tracking methods in primates and dogs. Before the development of eye-tracking methods, methods using the body (or face) direction or orientation were used in humans, primates, dogs, birds, and tortoises because they use binocular vision when they pay attention^6,27–30^. Fish also directed to the attention point. For example, females of guppy usually orient toward stimulus males^31^ and goldfish can detect a target more precisely when they see it in the binocular field^32^. Recently, in guppy, body orientation was used as a surrogate for viewing and inspecting the small target created by a laser pointer^33^. Because *N. brichardi* directed and fixated toward conspecific moving image^16^, we thought that body direction could be used for attention-tracking in *N. brichardi*. However, previous study showed that *N. pulcher* preferred their right eye to viewing their mirror images^34^. Therefore, similar to previous study in guppy^33^, we first examined how *N. brichardi* inspect a small target (Experiment 1). Since the result of Experiment 1 showed that subject fish oriented toward a small target and the body axis of fish can be used to estimate the point where *N. brichardi* is viewing or inspecting as an attention-tracking method (see Experiment 1), we tested whether *N. brichardi* paid attention to the face and examined the face-viewing pattern in *N. brichardi* using the attention-tracking methods from Experiment 1 (Experiment 2).

## Experiment 1

In Experiment 1, we explored whether the extended body axis of the fish passed through the point to which the fish was paying attention. To elicit attention from focal fish, we used a laser pointer to present a red spot^33^. Studies have used a laser pointer to attract the subject’s attention in African penguins^29^ and red-footed tortoises^28^. In some fish, the red spot created by a laser pointer was assumed to be real prey because subjects exhibited feeding-like behaviours^33,35^. Because *N. brichardi* feed plankton^36^, we expected that they paid attention to the small target. If *N*. *brichardi* stopped so that its body was directed toward the red spot, we could use the extended body axis of the fish to estimate where it was paying attention.

### Materials and methods

**Figure 1:**
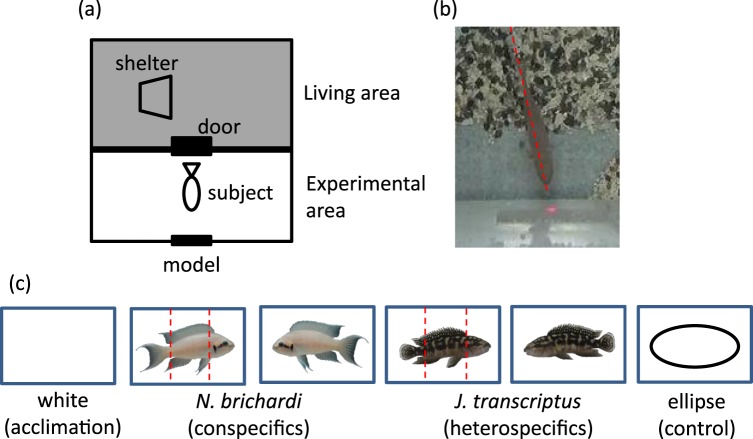
Experimental setup and stimulus cards. (**a**) Experimental apparatus. (**b**) Evaluation of the point toward which fish payed attention. (**c**) Stimulus cards: all stimuli were 4.3 cm wide.

*Neolamprologus brichardi* were obtained from commercial breeders and each fish was acclimatised in a tank (45 × 30 × 30 cm) maintained at 26 ± 1 °C under a 12 h:12 h light:dark cycle. The two experiments were conducted in the same type of tank. Each had two compartments separated by a black opaque partition: a living area including half of a flowerpot as a shelter and aeration, and an experimental area where stimuli were presented (Fig. 1a). The partition had a central transparent door that was kept open when no experiment was underway, allowing the fish to move freely between the compartments. To familiarise the fish with the stimulation, we repeatedly presented a white card at the presentation site (Fig. 1c). Food (TetraMin flakes, Spectrum Brands Japan, Japan) was provided in the living compartment twice a day.

### Experimental procedures

Male *N. brichardi* (54.8–76.8 mm SL, N = 9) were used. We used a laser pointer (KS-201, Maxell, Japan) to create an approximately 3-mm-diameter bright red spot. When the focal fish moved into the experimental area, the red spot was focused on a defined point on the white card. When the fish noticed the red spot, it directed its head toward the red spot and stopped in front of the card at various distances (see Video S1). This fixation with the head directed toward the red spot after finding the spot indicated that the fish paid attention to the spot. The fish’s reaction to the red spot was video-recorded (HDR-CX370; Sony, Japan) from above the tank and the fixations of the subject fish were converted into still images with ELAN ver. 4.9.2 software. We conducted 27 trials to analyse the fish position with respect to the red spot, an average of 2.5 trials per fish (1 to 5 times; see Table S1). Two independent raters, HT (rater 1) and KK (rater 2), drew the extended body axis on still images and recorded where the point intersected the white card as the estimation spot in a blind condition, without information about the location of the red spot and the ID of the focal fish. The white card was divided into three areas, i.e. left, centre, and right, and where the estimation spot was recorded (estimation area) to verify our estimation method. We also measured the lateral deviation from the spot to the extended line of the body axis, where positive values mean that the line deviated to the right, i.e. the red spot was presented in left visual field, and the distance from the red spot to snout of focal fish.

### Statistical analyses

The statistical analyses were performed with R ver. 3.1.1 software. We calculated the concordance and Fleiss’ Kappa coefficient (κ) to assess reproducibility between the estimation areas of the two raters and the real spot area. Fleiss’ κ > 0.80 indicates excellent agreement^37^. We also used the Spearman rank-correlation test to compare the lateral deviation of the two raters and the one-sampled Wilcoxon signed-rank test for each rater to test whether focal fish prefer using either visual field.

### Results and discussion

**Figure 2:**
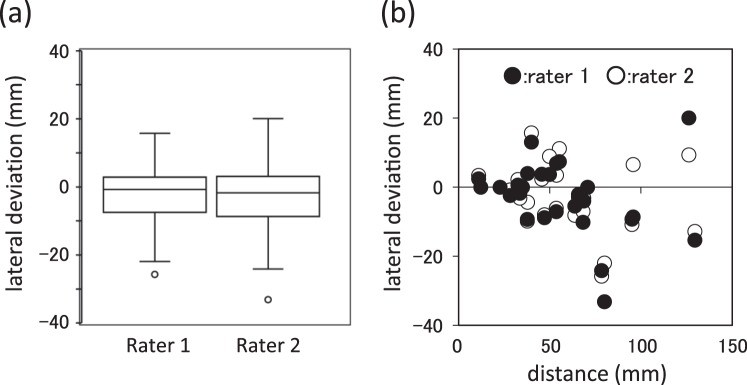
The results of Experiment 1. (**a**) Lateral deviations of each rater. (**b**) The relationships between lateral deviations of two raters and distance from real red spot to snout of fish. Black and white circles mean rater 1 and rater 2, respectively.

When a red spot was presented, the fish approached the spot rapidly (Fig. 1b, Video S1). This showed that subjects paid attention to the red spot similar to other fish. The concordance and Fleiss’ κ between the estimations of two raters and real spot area were 85.2% and Fleiss’ κ = 0.83, respectively, indicating excellent agreement^37^. The lateral deviations by the two raters were significantly correlated (Spearman rank-correlation test, r = 0.86, *p* < 0.001), and there was no evidence of using either visual field for each rater (rater 1, one-sampled Wilcoxon signed rank test, W = 91, *p* = 0.16; rater 2, W = 102, *p* = 0.29, Fig. 2a). The mean absolute lateral deviations from the spot to the extended line of the body axis (n = 27) by the two raters were 7.54 ± 7.95 (rater 1) and 6.95 ± 6.48 mm (rater 2). Furthermore, we did not find any relationship between the lateral deviation and the distance from the red spot to the snout of the focal fish (Fig. 2b, Table S1). These results indicate that *N. brichardi* directed to the red spot and the extended body axis of the fish accurately estimated where the fish was paying attention. Subsequently, we conducted Experiment 2 to test whether *N. brichardi* inspect to the face of other fish and examined the face-viewing pattern in *N. brichardi* using this estimation method.

## Experiment 2

In Experiment 2, to test whether *N. brichardi* inspect the face of other fish and examine the face-viewing pattern in *N*. *brichardi*, we presented subject fish with photographs of con- or heterospecifics. In recent years, computer-animated stimuli have been successfully used in several behavioural studies, including fish^38^. *N. pulcher* also showed adequate behavioural responses towards still stimulus images, i.e. con- and heterospecifics^39^, suggesting that methods using photographs are also valid for eliciting a response from *N. brichardi*. The face of *N*. *brichardi* includes much information, such as individual identity and fighting motivation, suggesting that they should initially pay attention to the face^25^. However, this might also occur because *N*. *brichardi* has clear colouration only on the face (Fig. 1c), which stands out in a photograph. Therefore, we provided a picture of the Lake Tanganyika cichlid, *Julidochromis transcriptus*, in which conspicuous colouration covers the entire body and the shape is different from *N. brichardi*^40,41^. *J. transcriptus* can also discriminate conspecifics using only facial pattern similar to *N. brichardi*^18^. If *N*. *brichardi* pays attention only to body parts that stand out in a fish photograph, the subject should pay attention to all regions of *J*. *transcriptus*. We also presented an ellipse to examine viewing pattern of non-fish stimuli (control). We predicted that *N. brichardi* would initially inspect the face of both conspecifics and heterospecifics, while the viewing pattern for the ellipse would be random.

### Materials and methods

Male *N. brichardi* (54.8–72.4 mm SL, N = 11) were used. The housing conditions of the experimental tank and familiarisation procedure with the white card were identical to those in Experiment 1.

### Experimental procedures

To prepare stimulus photographs, con- and heterospecific fish unfamiliar to the subject fish, were used. Lateral pictures of both sides of a fish in a small glass box (10 × 3.5 × 10 cm) were taken with a digital camera (IXY630; Canon, Japan) without anaesthetizing. The lateral images were transferred to a computer and the shape of each fish was cut out using GIMP ver. 2.8.10 software and pasted onto a white background measuring 6 × 4 cm [16, 18]. Then, the pictures were laminated. The stimulus size of the fish and ellipse was 4.3 cm (Fig. 1c), the same as in the previous study^16^.

The experimental tank was identical to that in Experiment 1. Before each experiment, when fish moved to the living area, the door was closed. Then, the white card was replaced with one of five stimulus cards (the right and left sides of *N. brichardi* and *J. transcriptus*, and the control ellipse; Fig. 1c). After 10 min, the door was opened and the fish was allowed to swim freely for 10 min, during which time its behaviour was video-recorded. The fish often fixated toward the stimuli card as in Experiment 1 (see Video S2). Then, the fixations of subject fish were converted into still images with ELAN ver. 4.9.2. One fish refused to enter the experimental area, and was excluded from the analysis, i.e. N = 10 for analyses. For 5 min after the fish entered the experimental area, we noted where it inspected, that is, where the extended body axis intersected the stimulus card (Fig. 1b) from still images. Then, we counted the number of fixation and measured the total time of fixation. The attention points were divided into three zones: the face, body, and tail of the test stimuli; and the left, centre, and right of the control ellipse. The stimulus cards were presented twice daily at an interval of at least 3 h, in pseudo-random order.

### Statistical analyses

The statistical analyses were performed with R ver. 3.1.1. We used a generalised linear mixed effect model (GLMM) to explore how *N*. *brichardi* viewed other fish. The number of fixation and total fixating time were fitted as Poisson and Gaussian response variables, respectively. The parts (face, body, or tail), species (*N*. *brichardi* or *J*. *transcriptus*), side of the model (left or right side) and three biological meaningful two-way interactions were fitted as potential explanatory variables. The final models were determined via backward elimination applying likelihood ration (LR) tests whereby all potential explanatory terms were entered into full models, and nonsignificant terms were removed from these models. At each step of elimination, we compared the reduced mode with the former model by LR tests. If there was significant difference between them, we decided the former models as the final models. Moreover, because we found significant effects for the parts (see Results). *Post hoc* tests were performed using GLMMs. In the *post hoc* GLMMs, the significance levels were adjusted using the Benjamini-Hochberg method^42^. For the control ellipses, only zone (left, centre, or right) was used as an explanatory variable. Although fish ID was included as random factor, we did not include the presentation order as a fixed effect in all models because there were too many explanatory variables. Additionally, we also added the AIC information in Table S4.

For this statistical test, we report the partial eta-square (ƞ^2^_p_) and Cohen’s d effect sizes to evaluate the meaningfulness of the differences because of our small sample size. In general, ƞ^2^_p_ values describe the amount of variance accounted for in the sample, where values of 0.01–0.03, 0.06–0.09, and >0.14 indicate a small, medium, and large effect, respectively^43^. The magnitude is also assessed using the thresholds, i.e. 0–0.2, 0.2–0.5, 0.5–0.8, and >0.8 indicate a negligible, small, medium, and large effect, respectively, using Cohen’s d value^44^.

We also measured how many of the first seven fixations were toward the face. Body parts that were fixated on the focal fish were fitted as a binomial response term (face or non-face body parts) in the GLMM where fish ID was fitted as a random effect. In this model, species, side of model, and fixation order were fitted as potential explanatory variables. Because all of the potential explanatory variables were rejected from the final model, we compared this number with that expected by chance (i.e. 33%), using a binomial test after all data were pooled.

To evaluate the reliability of data, another observer recorded where the fish paid attention (i.e. where the extended body axis intersected the stimulus card) in a blind condition. The inter-observer concordance was 100% and Cohen’s κ was 1.00 (Cohen’s κ > 0.75 indicates ‘excellent agreement^45^’).

### Results and discussion

**Figure 3:**
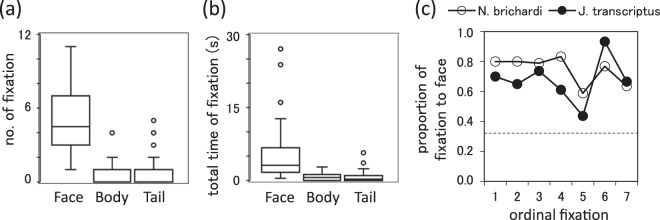
The results of Experiment 2. (**a**) Number of fixations. (**b**) Total fixation times (s). (**c**) Proportion of facial fixations. Open circles, responses to N. brichardi; filled circles, responses to J. transcriptus; dash line, the chance level (0.33).

In Experiment 2, species and side of models did not significantly affect the viewing pattern (p > 0.05 and ƞ^2^_p_ < 0.01 for all, these terms including interactions were removed from final model), suggesting that subjects paid attention to heterospecific pictures similar to conspecifics did not prefer using either eye when viewing the stimulus. However, when the stimulus card was presented, we found significant differences according to only the parts (number of fixations, χ^2^ = 229.2, ƞ^2^_p_ = 0.562, p < 0.001; total time of fixations, F = 12.17, ƞ^2^_p_ = 0.294, p < 0.001). Post-hoc test revealed that subject fish often oriented in direction to the face, for longer durations and many times than the body and tail [number: face vs. body, χ^2^ = 11.04, d = 3.71, *p* < 0.05, face vs. tail, χ^2^ = 142.63, d = 3.42, *p* < 0.05 (Fig. 3a); total time: face vs. body, F = 25.70, d = 2.11, *p* < 0.05, face vs. tail, F = 25.55, d = 2.10, *p* < 0.05 (Fig. 3b)]. There was no significant difference between body and tail [number: body vs. tail, χ^2^ = 1.27, d = 0.17, *p* = 0.204 (Fig. 3a); total time: body vs. tail, F = 0.02, d = 0.05, *p* = 0.89 (Fig. 3b)]. In the ellipse, we found no significant differences according to zone (number, χ^2^ = 0.02, ƞ^2^_p_ = 0.03, *p* = 0.88; total time, F = 1.06, ƞ^2^_p_ = 0.09, *p* = 0.33), indicating that fish directed to the non-fish stimulus at random. Figure 3c shows the time course of viewing during the first seven fixations. We confirmed that fish paid attention to the face of other fish both initially and during subsequent fixations [binominal test, *p* < 0.05 (Fig. 3c, see also Table S3)].

## General Discussion

This study explored a method for attention tracking in fish, similar to eye-tracking studies in primates (Experiment 1) and tested how fish view both con- and heterospecific fish (Experiment 2). The results of Experiment 1 showed that the extended body axis line closely approximated the attention point. These findings suggest that our method is appropriate for estimating where focal fish are paying attention, and is a good approach for evaluating fish attention-tracking. The results of Experiment 2 revealed that *N*. *brichardi* oriented toward the faces of conspecific fish in both initial and subsequent fixations, i.e. they paid more attention to the face than to other body parts, irrespective of the direction of model. This suggests that *N. brichardi* pay more attention toward face than other body parts.

By contrast, it is possible that *N. brichardi* paid more attention toward face because they have only salient coloration on face region. When humans viewed the pictures including face stimuli, they also tended to gaze on the saliency region other than face region^46^. To test this possibility, we also examined the viewing patterns toward *J. transcriptus*. Our result showed that subjects paid attention to the face of *J. transcriptus*, despite its striped colouration over its entire body^18,40,41^, similar to the case of the conspecific. Taken together, these results may show that *N*. *brichardi* pay more attention to the face of other fish rather than other body parts irrespective of saliency.

Our results support the idea that *N. brichardi* also pay more attention toward face than other body parts similar to humans and apes. This viewing pattern indicates that the face is informative and attractive regions and plays an important role in visual communication as in humans and apes^1–5,16–18,25,47^. Some have proposed a relationship between face-viewing and predator detection immediately after birth^7,48^. Young have little information about predator species and are more vulnerable to predators than adults, so natural selection would favour individuals capable of detecting predators before experiencing an attack^7^. Japanese monkeys *Macaca fuscata* preferred face stimuli more than non-face stimuli although they were reared without exposure to face^49^. Moreover, neonate chicks pecked at head more than other body parts^21^ and exhibited stronger filial preference toward the configuration of features associated with the head and necks even in the absence of previous experience^22^. In fish, total mouth size and eye distance are sufficient for good predator recognition^50^. In fact, naïve young of the reef fish *Chromis caeruleus* avoids the faces of typical piscivorous fish, in which the mouth has a concave shape and the eyes have wide vertical dimensions^51^, and juvenile African jewel fish fled from an approaching model with two black spots resembling facing eyes^48^, suggesting that fish can recognise a predator based only on the face and that face-viewing at first sight is beneficial for young fish to avoid predators. Thus, face-viewing at first sight has already evolved in fish for predator detection. To reveal this prediction, we should examine the face-viewing pattern of fish with no previous experience to face stimuli^49^.

Contrasting the similarities in face-viewing at first sight between primates and fish, our results revealed a difference in the face-viewing pattern. After the first attention, the face-viewing pattern appears to differ among species. For example, chimpanzees shift their fixation location more quickly and more broadly than humans probably because chimpanzees avoid direct eye contact^1,5^. *N*. *brichardi* paid attention to face at first, but continue to inspect for a longer period than do humans and apes^1,2^. Humans and apes need to look at other body parts, such as the arms and legs, to predict future movements or an object which focal individual is paying attention^52^. By contrast, fish use fins for locomotion, and movement cannot be predicted by evaluating the appearance of the fins. Fish generally move forward, i.e. direction of the head, and their movement can be predicted by only the direction of the head. When fish flee from predators, they use the C-start, which consists of a sudden turn toward the direction of flight^53^. Sneakers can also understand whether territory owners are aware of their existence from the direction of the head^54^. Thus, fish may need to check the direction of the head often after the first glance to gather much information, and *N*. *brichardi* persist in paying attention to the face. Thus, differences in face-viewing patterns after the initial fixation reflect variation in facial information.

The face-viewing pattern toward heterospecifics in *N*. *brichardi* also differs from that of primates and dogs. Although both the number and total time of fixation did not differ between con- and heterospecific models in *N*. *brichardi*, primates and dogs tend to gaze at conspecific faces longer and more frequently than at heterospecific faces^1,6,55^. The cause of the preference for conspecific faces is thought to be expertise in the perception of the faces of one’s own species. *N*. *brichardi* and *J*. *transcriptus* both live in a rocky habitat in Lake Tanganyika^24,40^ and they are observed to fight for limited shelter. In fact, some cohabiting Tanganyikan cichlid species form interspecific social relationships such as those involving interspecific territories^56,57^. Therefore, the faces of heterospecific fish also convey as much information as those of conspecifics for *N*. *brichardi*^16,18^. Some argued that the differences in gazing between con- and heterospecific may be explained by the fact that subjects did not require to collect information from face^2^. However, in this study, because we used unfamiliar fish for stimuli, it is possible that our subject fish required information from face and continued viewing. Further studies using familiar *J. transcriptus* or other allopatric fish will reveal whether the viewing pattern differs according to their relationship in natural habitat.

In Experiment 2, subject fish directed the face area irrespective of the side of models, suggesting that they did not prefer either eye to view the stimulus and the face part was presented in binocular visual field. This result is consistent with our previous study showing *N. brichardi* oriented toward a conspecific moving image^16^. Although many studies showed advantage of visual lateralization^58^, non-lateralized fish were found in natural populations in fish. In fact, Dadda *et al*.^59^ showed that less lateralized topminnows outperformed over lateralized fish in the task requiring to integrate the information from the left and the right visual field. This may indicate that subjects tended to gather much information from face by viewing with both eyes. However, other another study showed that *N. pulcher* preferred using their right eye when viewing a mirror image^34^. Because these studies did not use real fish as stimuli, further study on visual lateralization in *N. brichardi* will be needed.

This method of estimating the attention of fish has been applied in humans, primates, and dogs^1–3,6^. Some studies have suggested that the orientation behaviour may include both bouts of active information processing and blank stares^60^. We did not exclude this possibility because the focal fish stopped in directions not directed toward the stimulus card. In fact, a previous study on the optokinetic response of zebrafish showed the difference between body axis and eye axis^61^. Further study using eye-tracking and ray-tracing is needed to reveal the face-gazing pattern in fish.

Eye-tracking studies have revealed that humans and primates gaze first at the eyes and then scan the face^1–5^. Although *N*. *brichardi* pays attention first to the face, it remains unclear whether the eyes alone are the subject of the attention. However, several studies have shown that the fish eye is important in terms of species recognition and social status^62,63^, suggesting that fish might pay attention to the eyes rather rapidly at first, and then gather social information as do other face-viewing animals^1–5^. If fish engage in eye-viewing, this would render fish even more similar to humans and primates. Our methodology is applicable to the study of many other fish. Further research on face recognition by fish would help elucidate the evolution of social cognition in vertebrates.

### Ethics

All experiments were conducted in compliance with the Regulations on Animal Experiments of Osaka City University and the relevant dictates of the Japan Ethological Society. No permit from the Japanese Government is required for experiments involving *N*. *brichardi* or *J*. *transcriptus*.

## Supplementary information

Video S1

Video S2

Supplementary material

## Data Availability

The raw data for each individual fish has been made available in the electronic Supplementary Material to this publication.
