# Supplementary material for: Fish focus primarily on the faces of other fish
Source: Sci Rep. 2019 Jun 10;9:8377. doi: 10.1038/s41598-019-44715-0 (PMC6557811; doi:10.1038/s41598-019-44715-0)
Supplement: Supplementary file 3 — Supplementary material [file 41598_2019_44715_MOESM3_ESM.docx]

**Fish focus primarily on the faces of other fish**

Takashi Hotta, Kento Kawasaka, Shun Satoh, Masanori Kohda

**Supplementary Results**

Table S1. The raw data in Experiment 1. “Area” means a real and estimated area, i.e. left, center or right, on the white card, and “Distance” is the distance between the red spot and the top of snout of focal fish. “Difference” indicates the lateral distance from the spot to the extended line of the body axis (positive value means that the line deviated to the right, i.e. focal fish used the left eye).

| Fish ID | Order | Red spot | | Observer 1 | | Observer 2 | |
| --- | --- | --- | --- | --- | --- | --- | --- |
|  |  | Area | Distance (mm) | Area | Difference (mm) | Area | Difference(mm) |
| B21 | 1 | Right | 53.610 | Right | 3.466 | Right | 6.928 |
|  | 2 | Left | 37.780 | Left | -4.358 | Left | 3.955 |
|  | 3 | Center | 63.684 | Left | -8.103 | Left | -5.447 |
|  | 4 | Right | 126.296 | Right | 9.379 | Right | 20.078 |
|  | 5 | Right | 55.231 | Right | 11.168 | Right | 7.379 |
| B9 | 1 | Center | 78.369 | Left | -25.733 | Left | -24.114 |
| SM2 | 1 | Left | 68.154 | Left | -7.025 | Left | -10.125 |
|  | 2 | Center | 53.522 | Center | 6.059 | Center | -7.052 |
|  | 3 | Right | 49.824 | Right | 8.964 | Right | 3.736 |
| SM3 | 1 | Right | 65.651 | Right | -2.847 | Right | -2.106 |
|  | 2 | Center | 37.654 | Center | -3.156 | Center | -1.765 |
|  | 3 | Left | 33.573 | Left | -9.820 | Left | -9.316 |
| SM4 | 1 | Right | 28.280 | Right | -0.749 | Right | -2.371 |
|  | 2 | Right | 39.970 | Right | 15.714 | Right | 13.095 |
|  | 3 | Center | 10.966 | Center | 3.396 | Center | 2.481 |
|  | 4 | Left | 22.697 | Left | 0.000 | Left | 0.000 |
|  | 5 | Right | 12.176 | Right | 0.000 | Right | 0.000 |
| SM5 | 1 | Center | 68.558 | Center | -3.126 | Center | -4.019 |
|  | 2 | Right | 79.941 | Center | -21.941 | Left | -33.134 |
|  | 3 | Left | 47.022 | Left | -7.938 | Left | -8.805 |
|  | 4 | Center | 95.060 | Center | -10.787 | Center | -9.245 |
| B22 | 1 | Left | 45.615 | Left | 2.370 | Left | 3.760 |
| SM6 | 1 | Right | 70.766 | Right | 0.000 | Right | 0.000 |
|  | 2 | Left | 32.501 | Left | 2.260 | Left | 0.648 |
|  | 3 | Center | 34.982 | Center | 0.000 | Center | 0.000 |
| UK | 1 | Center | 95.688 | Right | 6.543 | Center | -8.635 |
|  | 2 | Center | 129.563 | Left | -12.799 | Left | -15.332 |

Table S2. The number and total time of fixation for each individual fish in Experiment 2. In the stimulus type, con, het, L and R indicate conspecific, heterospecific, left and right, respectively. For example, con-L is conspecific left side model. Although two of 10 fish were used repeatedly, the interval between experiments was more the three weeks.

| fish ID | stimulus type | order | the number of fixation | | | total time of fixation (s) | | |
| --- | --- | --- | --- | --- | --- | --- | --- | --- |
|  |  |  | Left | center | right | Left | Center | right |
| B4 | con-L | 3 | 1 | 0 | 0 | 0.492 | 0 | 0 |
|  | con-R | 4 | 0 | 0 | 2 | 0 | 0 | 1.635 |
|  | het-L | 1 | 5 | 0 | 1 | 7.279 | 0 | 0.267 |
|  | het-R | 5 | 0 | 0 | 2 | 0 | 0 | 1.635 |
|  | control | 2 | 1 | 0 | 2 | 0.334 | 0 | 0.735 |
| B5 | con-L | 1 | 7 | 0 | 0 | 6.685 | 0 | 0 |
|  | con-R | 5 | 2 | 2 | 3 | 1.350 | 2.136 | 2.9 |
|  | het-L | 4 | 7 | 0 | 0 | 9.479 | 0 | 0 |
|  | het-R | 3 | 1 | 1 | 4 | 1.232 | 1.177 | 4.999 |
|  | control | 2 | 3 | 4 | 3 | 2.480 | 1.827 | 5.017 |
| B9 | con-L | 5 | 4 | 1 | 0 | 2.768 | 0.334 | 0 |
|  | con-R | 4 | 0 | 0 | 4 | 0 | 0 | 2.136 |
|  | het-L | 2 | 5 | 0 | 1 | 2.099 | 0 | 0.434 |
|  | het-R | 3 | 1 | 1 | 2 | 0.234 | 0.734 | 0.968 |
|  | control | 1 | 0 | 2 | 1 | 0 | 0.787 | 0.134 |
| B10 | con-L | 5 | 3 | 0 | 0 | 2.546 | 0 | 0 |
|  | con-R | 1 | 1 | 1 | 7 | 0.865 | 2.156 | 23.824 |
|  | het-L | 3 | 5 | 0 | 1 | 3.486 | 0 | 0.588 |
|  | het-R | 4 | 1 | 1 | 6 | 0.660 | 0.718 | 6.487 |
|  | control | 2 | 4 | 4 | 4 | 4.078 | 1.112 | 7.276 |
| B15 | con-L | 1 | 10 | 2 | 3 | 11.534 | 2.001 | 1.602 |
|  | con-R | 5 | 2 | 0 | 5 | 1.200 | 0 | 4.498 |
|  | het-L | 2 | 8 | 1 | 4 | 6.750 | 0.476 | 2.411 |
|  | het-R | 3 | 1 | 2 | 6 | 0.200 | 0.805 | 3.285 |
|  | control | 4 | 1 | 2 | 0 | 0.502 | 1.655 | 0 |
| B16 | con-L | 2 | 11 | 2 | 0 | 11.986 | 2.750 | 0 |
|  | con-R | 4 | 0 | 2 | 4 | 0 | 2.170 | 2.303 |
|  | het-L | 1 | 7 | 1 | 0 | 16.046 | 0.634 | 0 |
|  | het-R | 5 | 1 | 0 | 1 | 1.915 | 0 | 1.454 |
|  | control | 3 | 1 | 1 | 1 | 0.699 | 0.949 | 0.723 |
|  |  |  |  |  |  |  |  | Continued |
| B18 | con-L | 4 | 3 | 0 | 0 | 1.752 | 0 | 0 |
|  | con-R | 5 | 1 | 0 | 5 | 0.317 | 0 | 2.091 |
|  | het-L | 2 | 1 | 1 | 1 | 0.433 | 1.356 | 0.823 |
|  | het-R | 1 | 1 | 2 | 3 | 0.378 | 1.010 | 4.049 |
|  | control | 3 | 1 | 1 | 1 | 1.248 | 1.622 | 3.459 |
| B19 | con-L | 2 | 7 | 1 | 0 | 4.740 | 1.161 | 0 |
|  | con-R | 1 | 0 | 1 | 3 | 0 | 0.734 | 5.172 |
|  | het-L | 4 | 3 | 1 | 0 | 2.661 | 0.909 | 0 |
|  | het-R | 5 | 5 | 2 | 7 | 5.961 | 2.757 | 27.052 |
|  | control | 3 | 2 | 7 | 3 | 0.964 | 8.809 | 6.738 |
| B21 | con-L | 5 | 8 | 0 | 2 | 2.850 | 0 | 0.701 |
|  | con-R | 4 | 1 | 4 | 3 | 0.367 | 2.035 | 1.268 |
|  | het-L | 1 | 2 | 1 | 0 | 0.934 | 0.467 | 0 |
|  | het-R | 2 | 2 | 1 | 4 | 1.035 | 0.667 | 1.548 |
|  | control | 3 | 5 | 5 | 5 | 5.973 | 3.803 | 3.725 |
| B23 | con-L | 2 | 7 | 1 | 1 | 12.676 | 0.367 | 0.276 |
|  | con-R | 4 | 3 | 1 | 5 | 3.947 | 0.600 | 4.239 |
|  | het-L | 3 | 5 | 1 | 0 | 10.967 | 1.234 | 0 |
|  | het-R | 5 | 2 | 1 | 3 | 3.585 | 1.942 | 1.231 |
|  | control | 1 | 2 | 0 | 1 | 1.803 | 0 | 1.461 |

Table S3. The order of fixation parts for each individual fish in Experiment 2. ‘F’, ‘B’ and ‘T’ show face, body and tail, respectively. (i) conspecific left side stimuli, (ii) conspecific right side stimuli, (iii) heterospecific left side stimuli and (iv) heterospecific right side stimuli.

| (i) | fixation order | | | | | | |
| --- | --- | --- | --- | --- | --- | --- | --- |
| fish ID | 1 | 2 | 3 | 4 | 5 | 6 | 7 |
| B4 | F | T | T | F | T | F |  |
| B5 | F | F | F | F | F | F | F |
| B9 | F | F | F | F | B | B |  |
| B10 | F | F | F | F | F | T | F |
| B15 | F | F | F | F | T | F | B |
| B16 | F | F | T | F | F | T | F |
| B18 | F | F | F |  |  |  |  |
| B19 | F | F | F | F | F | F | B |
| B21 | F | F | F | T | F | F | F |
| B23 | F | F | F | F | F | T | F |
| (ii) | fixation order | | | | | | |
| fish ID | 1 | 2 | 3 | 4 | 5 | 6 | 7 |
| B4 | F | F | F | F |  |  |  |
| B5 | F | T | B | B | F | T | F |
| B9 | F | F | F | F | F |  |  |
| B10 | F | F | F | F | F | F | F |
| B15 | F | T | F | T | F | F | F |
| B16 | B | F | F | F | F | B |  |
| B18 | T | F | F | F | F | F |  |
| B19 | F | F | F | F | F | F | F |
| B21 | T | F | B | F | F | B | B |
| B23 | T | T | F | B | F | F | F |
|  |  |  |  |  |  |  |  |
| (iii) | fixation order | | | | | | |
| fish ID | 1 | 2 | 3 | 4 | 5 | 6 | 7 |
| B4 | T | F | F | F | T | F | F |
| B5 | F | T | F | F | T | F | T |
| B9 | F | T | F | F | B | F | T |
| B10 | T | F | F | F | F | F | F |
| B15 | F | F | F | T | F | F | F |
| B16 | F | F | F | F | F | F | F |
| B18 | B | T | F |  |  |  |  |
| B19 | B | F | F | F |  |  |  |
| B21 | F | B | B | F | T |  |  |
| B23 | F | F | F | F | B | F |  |
|  |  |  |  |  |  |  |  |
| (iv) | fixation order | | | | | | |
| fish ID | 1 | 2 | 3 | 4 | 5 | 6 | 7 |
| B4 | F | F | F | F | F | F | F |
| B5 | F | F | F | B | B | F | T |
| B9 | F | B | F | T |  |  |  |
| B10 | F | F | F | F | F | F | F |
| B15 | F | F | B | F | T | F | B |
| B16 | T | F |  |  |  |  |  |
| B18 | F | F | T | B | B | F |  |
| B19 | F | T | T | B | B | F | F |
| B21 | T | F | F | T | F | B | F |
| B23 | F | T | B | T | F | F |  |

Table S4. The AIC information and the AIC difference from the lowest model (ΔAIC). The 5 lowest models were described. (a) the number of fixation (b) the total duration of fixation.

| (a) |  |  |
| --- | --- | --- |
| model | AIC | ΔAIC |
| number = direction * parts | 382.40 | - |
| number = direction * parts + species | 384.02 | 1.62 |
| number = direction * parts + species * parts | 385.75 | 3.34 |
| number = parts | 386.70 | 4.29 |
| Full model | 387.37 | 4.97 |
|  |  |  |
| (b) |  |  |
| model | AIC | ΔAIC |
| Full model | 647.21 | - |
| time = direction * parts + direction * species | 648.57 | 1.36 |
| time = direction * parts + species | 648.95 | 1.74 |
| time = parts | 649.25 | 2.04 |
| time = parts * species | 650.22 | 3.01 |

**Supplementary Videos**

Video S1. An example of fixation toward a red spot.

Video S2. An example of fixation when conspecific stimuli card (con-R, i.e. face was right) was presented.
